# Editorial: The role of galectins in the immune microenvironment in human cancers and potential therapeutic approaches

**DOI:** 10.3389/fimmu.2024.1448407

**Published:** 2024-06-24

**Authors:** Md Zahidul Islam Pranjol, Dmitry Aleksandrovich Zinovkin, Michael Petrovich Potapnev

**Affiliations:** ^1^ Department of Biochemistry & Biomedicine, School of Life Sciences, University of Sussex, Brighton, United Kingdom; ^2^ Department of Pathology, Gomel State Medical University, Gomel, Belarus; ^3^ Department of Cellular Biotechnologies, Republican Scientific and Practical Center for Transfusiology and Medical Biotechnologies, Minsk, Belarus

**Keywords:** galectins, tumour microenvironment, immunosuppression, immunotherapy, therapeutics

## Introduction

Galectins, a family of β-galactoside-binding proteins, have emerged as key modulators, influencing the tumour microenvironment and immune evasion mechanisms. Despite not being classified as primary oncogenic drivers, recent research highlights their significant role in cancer progression, particularly through changing of the tumour and immune system interactions.

Galectins facilitate tumour immune escape by reducing immune cell infiltration into the tumour core, primarily by remodelling the tumour stroma and extracellular matrix (ECM). This results in immune-excluded tumours where lymphocytes recognise antigens but fail to penetrate the tumour nodules. The inhibition of galectins has shown promise in pre-clinical models, particularly when combined with other therapies such as immune checkpoint inhibitors, CAR-T cells, and anti-tumour vaccines.

This editorial synthesises insights from eight recent publications exploring galectins’ diverse roles in cancer biology. From their involvement in immunosuppressive tumour microenvironments in prostate and pancreatic cancers to their modulation of immune responses in rare cancers, these studies underscore the therapeutic potential of targeting galectins. Novel inhibitors like LLS30 and GB1211 demonstrate promising efficacy in enhancing anti-tumour immunity and improving the effectiveness of existing cancer therapies.

## Summary of contributions in this research topic

### Galectins: gatekeepers of the tumour microenvironment

In the context of cancer, galectins’ role becomes particularly sinister as they facilitate tumour progression and immune escape. The review article by Kapetanakis and Busson underscores the significance of galectins in oncogenesis and immune exclusion, highlighting their dual role in promoting tumour growth and inhibiting effective anti-tumour immune responses. Galectins remodel the tumour stroma and extracellular matrix, reducing immune cell infiltration and creating immune-excluded tumours. This makes them a crucial target for developing therapeutic agents. Despite promising pre-clinical and early clinical results, galectin inhibitors have yet to be fully explored in clinical settings. Profiling tumour and circulating galectins could enhance cancer treatment strategies by combining galectin inhibition with other therapies.

### Modulating T cell function in prostate cancer

One of the key studies in this Research Topic, a research article by Shih et al., focuses on prostate cancer, demonstrating that targeting galectin-1 can significantly improve cancer immunotherapy outcomes. The study explores Galectin-1 (Gal-1) in the immunosuppressive tumour microenvironment (TME) of prostate cancer (PCa). Elevated Gal-1 expression correlates with advanced PCa stages and induces T cell apoptosis. Gal-1, secreted by PCa cells, contributes to tumour progression by promoting T cell apoptosis. Inhibiting Gal-1 with LLS30 increases intratumoral T cell infiltration and enhances anti-PD-1 therapy efficacy. LLS30 binds to the carbohydrate recognition domain of Gal-1, preventing its interaction with CD45 and thus suppressing T cell apoptosis. RNA-seq analysis links LLS30’s effects to anti-tumour immunity, suggesting a potential therapeutic strategy.

### Hypoxia and immunosuppression in pancreatic cancer

The relationship between hypoxia and immunosuppression is well-documented. Pancreatic ductal adenocarcinoma (PDAC) is notably aggressive and often resistant to treatment, partly due to tumour hypoxia, which creates an immunosuppressive microenvironment. Using the Buffa hypoxia score, Sadozai et al. profiled PDAC cases, revealing that high hypoxia correlates with advanced tumour grade and reduced survival. High hypoxia tumours exhibited fewer T cells, NK cells, and dendritic cells, and elevated immunosuppressive molecule mRNA levels. Galectin-3 (LGALS3) emerged as a key gene associated with hypoxia. These findings suggest targeting hypoxia and associated immune molecules could enhance PDAC immunotherapy.

### Galectins in rare cancers

Diaz-Alvafez et al. reviews galectins’ roles in rare cancers which remains poorly understood, with conflicting evidence regarding their impact on prognosis and tumour growth. Understanding galectin interactions, such as glycan-independent binding to chemokines, is crucial. For example, Galectin-1 (Gal-1) binding to CXCL4 modifies its carbohydrate-binding site, increasing apoptosis in activated CD8+ T cells. Conversely, Galectin-9 (Gal-9) interaction with CCL5 reduces apoptosis in CD4+ T cells. These findings highlight the importance of considering galectin interactions within the tumour microenvironment.

### Intracellular galectins: new frontiers

Intracellular galectins, often overlooked in cancer research, are brought to the fore in the review by Nehme and St-Pierre. The review focuses on the emerging significance of intracellular galectins in cancer progression. While extracellular galectins’ roles in tumour growth and metastasis are well-studied, intracellular galectins, residing in cytosolic and nuclear compartments, interact with various ligands to modulate cellular processes. These interactions influence gene expression, signalling pathways, and tumour development. Targeting intracellular galectins using small inhibitors, antisense-oligonucleotide and siRNA drugs, peptides and intrabodies present promising therapeutic strategies, though challenges remain due to their complex biology and redundancy. Future research aims to better understand their roles and develop effective inhibitors.

### Anti-galectin-9 immunotherapy in pancreatic cancer

Another promising therapeutic approach is highlighted in the research article by Quilbe et al., which investigates a novel anti-galectin-9 immunotherapy in pancreatic cancer. Pancreatic adenocarcinoma (PDAC) often resists immune checkpoint inhibition due to its highly immunosuppressive microenvironment, characterised by an abundance of regulatory T cells (Tregs). To counteract this, researchers developed a monoclonal antibody targeting galectin-9 (LGALS9), crucial for Treg-mediated immunosuppression. Their study demonstrated that this anti-LGALS9 antibody effectively limits the progression of pancreatic neoplastic lesions in transgenic mice by reducing LGALS9 expression and Treg activity. This novel approach opens new avenues for immunotherapy in PDAC, highlighting the potential of targeting galectin-9 to improve treatment outcomes.

### Overcoming resistance to PD-1/PD-L1 blockade

Galectin-3 (Gal-3) is a β-galactoside-binding lectin highly expressed in the tumour microenvironment of aggressive cancers, often predicting poor response to pembrolizumab. Mabbitt et al. investigated if Gal-3 directly interacts with the PD-1/PD-L1 complex, potentially inhibiting immune checkpoint therapy. *In vitro* and *in vivo* assays showed Gal-3 enhances PD-1/PD-L1 interaction, reducing the efficacy of pembrolizumab and atezolizumab. However, using the Gal-3 inhibitor GB1211 restored these therapies’ binding efficiency, reduced tumour growth, and increased tumour-infiltrating T lymphocytes, suggesting GB1211 as a promising adjunct to checkpoint inhibitors.

### Biological challenges of galectin inhibition

Galectin inhibitors, promising for cancer therapy, face several clinical challenges as reviewed by Laderach and Compagno. These include ensuring high affinity and specificity, as galectins have similar structures and multiple isoforms. Effective inhibition requires understanding galectin functions and their role in cancer versus normal physiology. Pharmacokinetics, biodistribution, and resistance development are critical considerations. Improved inhibitor designs should resist degradation and selectively target tumours without affecting healthy tissue. Addressing these challenges could enhance the clinical application of galectin inhibitors for cancer treatment.

## Conclusion

The expanding field of galectin research offers promising advancements for cancer therapy. Galectins modulate the tumour microenvironment with focus on immune component, playing key roles in tumour progression and immune evasion. By understanding these mechanisms, we can develop more effective, focused mostly on cancer tissues personalised treatments. This Research Topic opens new avenues for combining galectin inhibitors with other cancer therapies, enhancing overall treatment efficacy. As our understanding deepens, the prospects for innovative and precise cancer therapies become increasingly hopeful.

## Author contributions

MZP: Conceptualization, Project administration, Supervision, Writing – original draft, Writing – review & editing. DZ: Writing – review & editing. MPP: Writing – review & editing.

